# Endoscopic Ultrasound in Guiding Local Resection and Ampullary Preservation of a High-Risk Periampullary GIST

**DOI:** 10.1155/2020/8418905

**Published:** 2020-01-30

**Authors:** Andrew Spiel, Ravi Patel, Rebecca Minter, Amir Ali Rahnemai Azar, Rashmi Agni, Antonio Bosch, Deepak Gopal

**Affiliations:** ^1^Division of Gastroenterology & Hepatology, Department of Medicine, Univeristy of WIsconsin School of Medicine and Public Health, Madison, WI, USA; ^2^Division of Surgical Oncology, Department of Surgery, University of Wisconsin School of Medicine and Public Health, Madison, WI, USA; ^3^Department of Pathology & Laboratory Medicine, University of Wisconsin School of Medicine and Public Health, Madison, WI, USA

## Abstract

Gastrointestinal stromal tumors (GISTs) typically develop in the stomach or small intestine and rarely involve the ampulla of Vater, with only 13 cases reported in the world literature since 2004. Most authors advocate performing pancreaticoduodenectomy for such lesions. However, this operation can carry higher rates of morbidity and mortality compared to local resection. We present a case of a high-risk, invasive periampullary GIST and the multidisciplinary management approach to local resection with the aid of endoscopic ultrasound. In addition, this case shows no local recurrence at 3 months and a favorable clinical outcome at 1 year.

## 1. Introduction

The ampulla of Vater can be home to a variety of neoplasms including adenomas, adenocarcinomas, choledochocele (type III), and neuroendocrine tumors [1]. Rarely, do these lesions represent gastrointestinal stromal tumors (GISTs). A GIST is generally defined as a spindle-cell tumor of the GI tract originating from the cells of Cajal (an intestinal pacemaker cell) [2]. By definition, they express *KIT* protein (CD117), which is a tyrosine kinase receptor protein [3]. Malignant potential of GIST is defined by size and mitotic index [4]. GISTs typically develop in the stomach or small intestine and rarely involve the ampulla of Vater, with only 13 cases reported in the literature [5]. Most authors advocate performing pancreaticoduodenectomy (PD) for such lesions [6]. Endoscopic ultrasound (EUS) can aid in surgical planning for local resection. We present a case of a high-risk, invasive periampullary GIST and the multidisciplinary management approach to local resection with preservation of the biliary tree and ampullary complex.

## 2. Case

**Figure 1:**
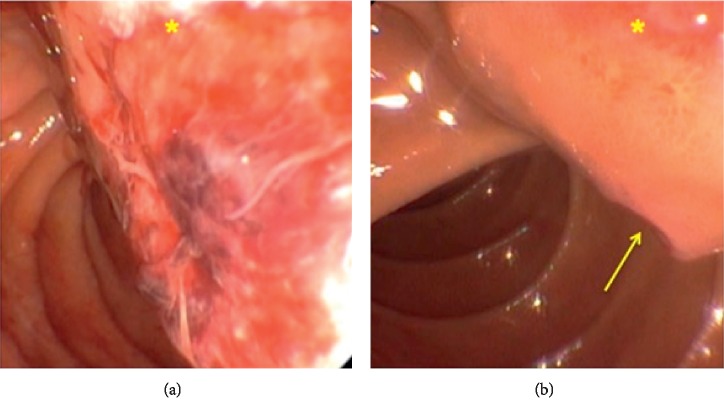
(a, b) Endoscopy. Ulcerated mass (^*∗*^) adjacent to ampulla of Vater (arrow).

**Figure 2:**
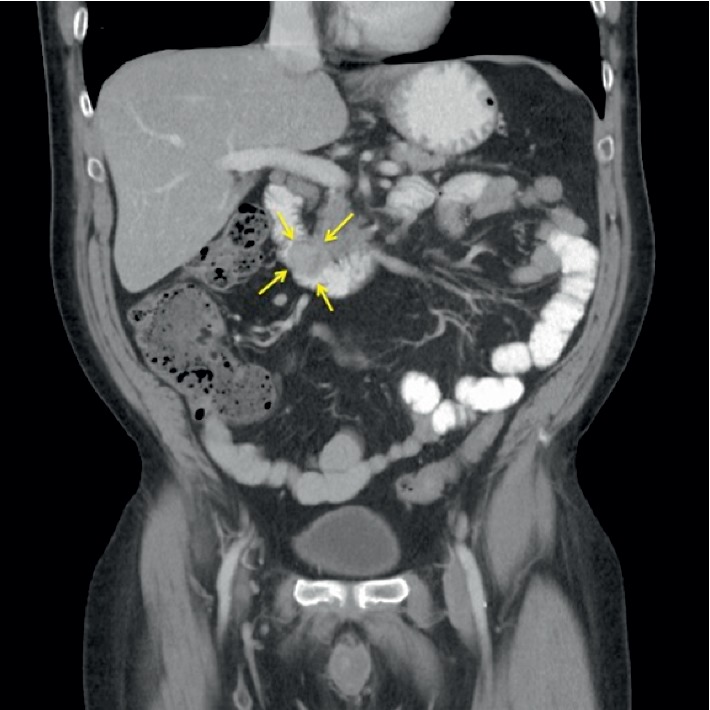
CT imaging showing a 2.6 × 1.4 cm intraluminal mass involving the proximal duodenum extending to the level of the ampulla.

**Figure 3:**
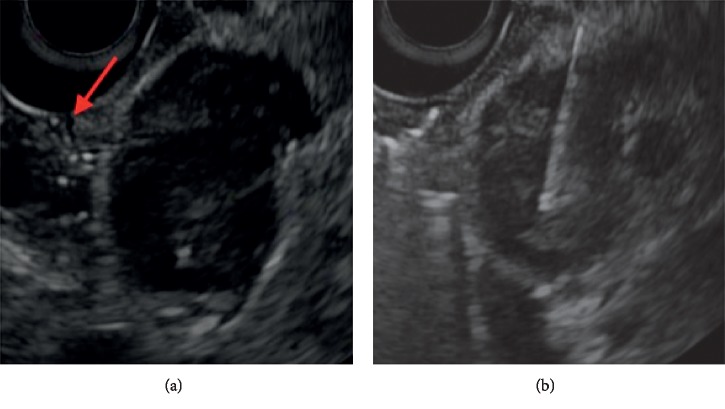
EUS. (a) Tumor extending from muscularis propria with identification of the papillary orifice (red arrow). Fine-needle biopsy of tumor (b).

**Figure 4:**
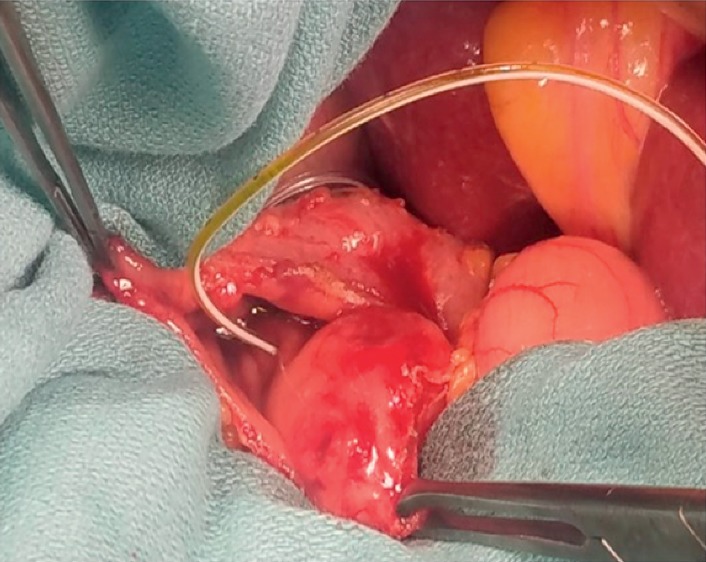
Intraoperative resection. Biliary catheter exiting ampulla.

**Figure 5:**
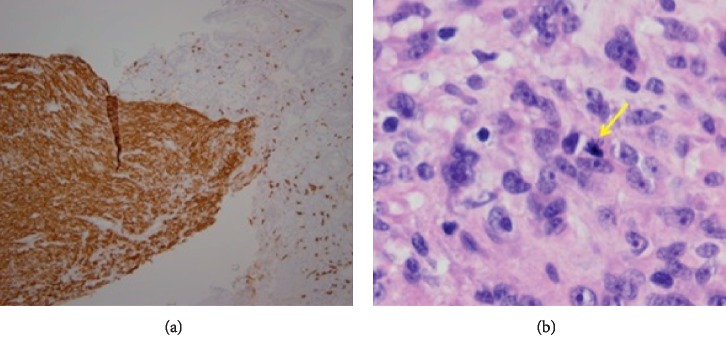
Pathology results. CD117-positive GIST (a). Mitotic body (arrow) 8 per 5 mm^2^ (high risk) (b).

A 61-year-old male presented to his primary care provider (PCP) for medication management. He had a history of chronic back pain, depression, anxiety, and peptic ulcer disease (PUD). On routine laboratory testing, he was noted to be anemic with a hemoglobin of 8.8 g/dL and, upon further questioning, endorsed 2 weeks of melena. Repeat Hgb was 9.2 g/dL, iron, 17 ug/dL, and ferritin, 6 ng/mL. Upper endoscopy revealed a 2.5 cm submucosal lesion with a central ulceration adjacent to the ampulla of Vater (Figures 1(a) and 1(b)). Standard forceps biopsy was obtained which revealed CD117- and DOG1-positive spindle-cell subtype GIST but was insufficient for risk assessment. CT of abdomen and pelvis was read as 2.6 × 1.4 cm intraluminal mass involving the proximal duodenum extending to the level of the ampulla (Figure 2). Endoscopic ultrasound (EUS) was performed showing a hypoechoic round mass in the area of the major papilla extending from the mucosa to the muscularis propria and measuring 2.5 cm by 1.6 cm in maximum cross-sectional dimensions. Biliary and pancreatic ducts were of normal caliber, and the papillary orifice was identified (red arrow) (Figure 3(a)). A core biopsy was obtained with a 22-gauge fine-needle biopsy (SharkCore™, Medtronic) (Figure 3(b)). EUS and side-viewing duodenoscope assisted in demonstrating that there was no involvement of the distal bile duct or head of the pancreas. Liver chemistry evaluation at that time showed total bilirubin of 0.6 mg/dL, AST of 29 U/L, ALT of 22 U/L, and alkaline phosphatase of 103 U/L. Core biopsy histopathology confirmed GIST but again was unable to further risk stratify by mitotic index. EUS images were reviewed with the pancreatobiliary surgeons in order to guide an operative plan. The preoperative distance of the tumor to the major papilla estimated <4 mm (approximated at 3-4 mm on EUS imaging), and the tumor deemed periampullary. The patient was therefore planned for surgery preparing for local excision based on EUS findings and normal liver chemistries, suggesting no invasion by the tumor of the adjacent pancreaticobiliary structures. Laparotomy was performed, and palpation confirmed that the ampullary complex and tumor capsule were thinly separated, on the order of millimeters as suggested by EUS. This allowed for completion of a duodenal excision with complex reconstruction, pyloric exclusion, and Billroth II jejunostomy (Figure 4) instead of a pancreaticoduodenectomy. Tumor capsule remained intact; a small portion of pancreatic head was resected due to extrinsic tumor adherence. Duodenal reconstruction involved 2 layers of suture to bring the cut edges of the duodenum together within the head of the pancreas. Final pathology showed a mixed-subtype, high-risk GIST staining positive for CD117 (Figure 5(a)) and 8 mitotic bodies per 5 mm^2^ (Figure 5(b)). Negative margins were obtained. His hospitalization was complicated by gastrojejunal anastomotic ulcer bleed and acute kidney injury. He underwent upper endoscopy with control of bleeding with full recovery. The patient's renal function returned to normal prior to discharge. Imatinib adjuvant chemotherapy was initiated, and he is currently tolerating with a brief treatment break due to lower extremity edema and fatigue in the first month. Surveillance imaging 3 months after resection shows no evidence of recurrence. 12 months after resection, he is clinically well and tolerating imatinib therapy.

## 3. Discussion

An ampullary or periampullary GIST is an extremely rare neoplasm. The majority of GISTs are located in the stomach (60%–70%) and the small intestine (20%–25%), with only 4% occurring in the duodenum [6]. These tumors classically arise from the cells of Cajal, and malignant potential is largely based on mitotic index >5/50 hpf and size [4].

Complete resection with negative margins carries the highest potential for cure of GIST [7]. Universally, surgical resection should be the approach to the treatment of these tumors when feasible. Lymphatic spread remains exceedingly rare, and lymphadenectomy is not routinely warranted. Many authors still advocate for pancreaticoduodenectomy (PD or Whipple Procedure) for treatment of these tumors, which are adjacent to or involve the ampulla of Vater. However, PD carries significant morbidity and mortality [8].

Of the 13 cases of ampullary GIST available for comparison in the literature, 3 were locally resected [5, 9, 10]. Surgical decision-making and feasibility of resection were aided by use of endoscopic ultrasound in 2 of these cases. This is the third case where local resection of a periampullary GIST is aided by use of EUS preoperatively.

Additionally, the utility of EUS with fine-needle aspiration or core biopsy to make a preliminary diagnosis is instrumental in surgical planning. Neuroendocrine tumors (NETs) and GISTs can appear similarly on endoscopy and imaging. Differentiation is critical with regard to surgical approach, as there is typical indication for lymphadenectomy in the case of NET [11]. The yield of standard forceps biopsy of subepithelial lesions is typically <10% and as low as 4% in one study [12].

A 2008 study by Goh et al. suggests similar disease-specific survival with local resection compared to PD for duodenal GIST's [13]. Of the total 7 patients who underwent local resection in this study, the median disease-specific survival was 144 months. Given the EUS findings of preserved ductal anatomy, our patient was referred and underwent successful local resection of the GIST with sparing of the ampullary complex.

Ultimately, this case demonstrates how local resection can be achieved with a multidisciplinary approach and use of EUS. This approach can lead to preservation of the ampullary and biliary complex while achieving negative margins in a high-risk periampullary GIST. Prior to referral for PD of a high-risk periampullary GIST, the authors would advocate for the use of EUS in confirming the diagnosis and consideration of local resection.
